# Pediatric Airway Assessment Tool (PAAT): A Rating Tool to Assess Resident Proficiency in Simulated Pediatric Airway Skills Performance

**DOI:** 10.15766/mep_2374-8265.10997

**Published:** 2020-10-19

**Authors:** Robyn Wing, Janette Baird, Susan Duffy, Linda Brown, Frank Overly, Mariann Nocera Kelley, Chris Merritt

**Affiliations:** 1 Assistant Professor, Departments of Emergency Medicine & Pediatrics, Division of Pediatric Emergency Medicine, Alpert Medical School of Brown University and Rhode Island Hospital/Hasbro Children's Hospital; Director of Pediatric Simulation, Lifespan Medical Simulation Center; 2 Associate Professor, Department of Emergency Medicine and Injury Prevention Center, Alpert Medical School of Brown University; 3 Professor, Departments of Emergency Medicine & Pediatrics, Division of Pediatric Emergency Medicine, Alpert Medical School of Brown University and Rhode Island Hospital/Hasbro Children's Hospital; 4 Associate Professor, Departments of Emergency Medicine & Pediatrics, Division of Pediatric Emergency Medicine, Alpert Medical School of Brown University and Rhode Island Hospital/Hasbro Children's Hospital; Vice Chair of Pediatric Emergency Medicine; Director of the Lifespan Medical Simulation Center; 5 Professor, Departments of Emergency Medicine & Pediatrics, Division of Pediatric Emergency Medicine, Alpert Medical School of Brown University and Rhode Island Hospital/Hasbro Children's Hospital; Medical Director of Hasbro Emergency Department; 6 Assistant Professor, Departments of Pediatrics and Emergency Medicine/Traumatology, Division of Pediatric Emergency Medicine, University of Connecticut School of Medicine, Connecticut Children's Medical Center; Director of Simulation Education, University of Connecticut School of Medicine; 7 Associate Professor, Departments of Emergency Medicine & Pediatrics, Division of Pediatric Emergency Medicine, Alpert Medical School of Brown University and Rhode Island Hospital/Hasbro Children's Hospital; Director, Brown Emergency Medicine Medical Education Research Fellowship

**Keywords:** Pediatric Airway, Simulation, Physician, Emergency Medicine, Pediatric Emergency Medicine, Pediatrics, Clinical Skills Assessment/OSCEs, Clinical/Procedural Skills Training, Assessment

## Abstract

**Introduction:**

The Accreditation Council for Graduate Medical Education has identified the need for assessment of core skills for pediatric and emergency medicine residents, which includes pediatric airway management. Although there are standard courses for pediatric airway management, there is no validated tool to assess basic and advanced pediatric airway skills performance. Our objective was to develop a simulation-based tool for the formative assessment of resident pediatric airway skills performance that was concise, yet comprehensive, and to evaluate the evidence supporting the argument for the tool's validity.

**Methods:**

We developed a pediatric airway assessment tool (PAAT) to assess six major domains of pediatric airway skills performance: basic airway maneuvers, airway adjuncts, bag-valve mask ventilation, advanced airway equipment preparation, direct laryngoscopy, and video laryngoscopy. This tool consisted of a 72-item pediatric airway skills assessment checklist to be used in simulation. We enrolled 12 subjects at four different training levels to participate. Assessment scores were rated by two independent expert raters.

**Results:**

The interrater agreement was high, ranging from 0.92 (adult bagging rate) to 1 (basic airway maneuvers). There was a significant trend of increasing scores with increased training level.

**Discussion:**

The PAAT demonstrated excellent interrater reliability and provided evidence of the construct's validity. Although further validation of this assessment tool is needed, these results suggest that the PAAT may eventually be useful for assessment of resident proficiency in pediatric airway skills performance.

## Educational Objectives

By the using this assessment, facilitators will be able to evaluate learners:

1. Demonstrating basic airway skills maneuvers.
2. Describing and properly using basic airway equipment such as the oropharyngeal airway, nasopharyngeal airway, and bag valve mask (BVM).
3. Demonstrating effective BVM ventilation on infant and adult patients.
4. Preparing for endotracheal intubation.
5. Demonstrating direct laryngoscopy and video laryngoscopy (Glidescope) for endotracheal intubation of infant and adolescent/adult patients.

## Introduction

In teaching hospitals, residents are often called to respond to children with respiratory failure, and must be proficient in basic airway management, and may be called upon to perform more advanced airway skills with supervision. However, residents are exposed to relatively few critically ill children, so must rely on alternative methods of developing proficiency in airway skills performance.^1^ Pediatric advanced life support (PALS) courses and other traditional learning methods have variable effects on proficiency.^2,3^ Knowledge retention and skill performance, including airway management, are reported to deteriorate quickly if not used.^4–6^ Medical simulation can be utilized to teach pediatric airway skills and to assess residents' abilities.^7–9^ The Accreditation Council for Graduate Medical Education (ACGME) has mandated the assessment of specific core skills, including emergency management and critical care procedures,^10^ and advocates for the use of simulation for both instruction and assessment.^11,12^ Recommendations that serve as a guide for the implementation of simulation-based assessments across the domains of validity, reproducibility, feasibility, educational and catalytic effects, have recently been published.^13^

There is currently no complete formative assessment tool for basic and advanced pediatric airway skills performance with sufficient evidence of validity. There have been other published tools for assessment of pediatric airway skills, but these were missing important components such as airway adjuncts and video laryngoscopy.^14–17^ A recent publication referenced a comprehensive airway assessment checklist for adult patients, but did not include a pediatric assessment and included no validity evidence.^18^ Given the significant anatomic differences and equipment choices for use in pediatric patients, this adult assessment was not suitable to assess providers skills in pediatric patients. Our objective was to develop a comprehensive simulation-based tool for the formative assessment of resident pediatric airway skills performance and to evaluate the evidence for the tool's validity. The construct being measured was an individual resident's ability to manage the airway of a patient in respiratory distress or failure. This tool's intended use was to assess whether residents possess the minimum basic skills to perform critical airway management under direct expert supervision during actual clinical encounters. Our goal was to use this tool as a formative assessment to provide feedback on current learner performance and help the learner to improve prior to performing these skills in a clinical setting. This formative assessment was one step in the process of preparing learners for eventual independent performance of the task.

We present our tool, the pediatric airway assessment tool (PAAT), and the results of a pilot study. This tool will advance assessment practice by allowing for standardized assessment of these critical skills in a safe learning environment prior to allowing learners to perform these activities in clinical practice.

## Methods

### Tool Development

Our team of pediatric emergency medicine specialists developed the PAAT after extensive literature review using the PALS curriculum^19^ and standard pediatric airway management knowledge. This tool consists of a 72-item observational checklist (Appendix A), including assessment of six domains of pediatric airway skills performance: basic airway maneuvers (three items), airway adjuncts (seven items), bag valve mask (BVM) ventilation (eight items), advanced airway equipment preparation (10 items), direct laryngoscopy (22 items), and video laryngoscopy (22 items). These six domains were categorized into three sections (ventilatory management skills, infant advanced airway, and adolescent advanced airway) on the checklist for feasibility of testing using specific airway equipment, task trainers, and manikins. Checklist items were chosen to assess critical actions in airway skills performance. The PAAT differs from previously published airway assessments in that it is concise yet comprehensive, including assessments of BVM, airway adjuncts, direct laryngoscopy, and video laryngoscopy on both infant- and adolescent-sized manikins.

A panel of 10 experts in emergency medicine, critical care, and medical education reviewed the tool for content, and to maximize the likelihood that performance of the critical actions measured in the test setting could be extrapolated to performance in clinical practice. Reviewers confirmed that questions and checklist items were accurate and expected for competent pediatric airway skills performance, eliminated inappropriate items, and added any necessary missing items. Modifications to the response process were made based on this feedback. Checklist items were carefully worded to ensure standardization of assessment and facilitate scoring, with scripted prompts to be used by the rater whenever possible. Two physician raters (who were part of the tool development team) trained for familiarity with the checklist and were directed to follow the assessment tool explicitly when rating the performance of the subjects enrolled in the pilot study.

The equipment needed included:

• Manikins:
  ○ SimMan (or other adolescent/adult-sized manikin that allowed for placement of airway adjuncts, endotracheal intubation, and assessment of chest rise with BVM)
  ○ SimBaby (or other infant-sized manikin that allowed for placement of airway adjuncts, endotracheal intubation, and assessment of chest rise with BVM)
• Equipment:
  ○ Stethoscope
  ○ Towels
  ○ BVMs with masks—multiple sizes∗
  ○ Nasopharyngeal airways—all sizes∗
  ○ Oropharyngeal airways—all sizes∗
  ○ Glidescope with various size handles∗
  ○ Glidescope stylet
  ○ Laryngoscope blades—multiple sizes∗
  ○ Endotracheal Tubes—multiple sizes∗
  ○ Stylets
  ○ 5–10 mL syringes
  ○ End-tidal CO_2_ detector/continuous CO_2_ monitor

∗The equipment should be appropriately sized for the manikins and reflective of the equipment used in local clinical practice.

### Pilot Subject Enrollment and Sessions

We recruited a convenience sample of 12 volunteer participants of different training levels (equal numbers of medical students, residents, pediatric emergency medicine fellows, and pediatric emergency medicine attending physicians) to pilot the assessment tool. Subjects gave verbal informed consent and completed a pretest to assess their pediatric airway management experience and self-efficacy with regard to pediatric airway skills. Subjects then entered the simulation lab where two high-fidelity simulation manikins (Laerdal SimBaby and SimMan, Laerdal Corporation) were set up on stretchers. All airway equipment, as listed above, was displayed for use and the participant was expected to choose the proper size equipment for the mannikin, as part of demonstrating their skill level in pediatric airway management. Participants performed the tasks listed in the pediatric airway skills assessment checklist (Appendix A) individually with scripted prompting from our raters and were instructed to verbalize their actions. Raters asked the participants to perform tasks using the outlined prompts in the PAAT, such as, “Demonstrate how to properly position and open the airway of this patient presenting in respiratory distress.” Raters were instructed to immediately remediate the participant prior to proceeding to the next section if they did not successfully complete an item. This remediation was initially done with additional scripted prompting, such as, “Is there anything else that you can do with your hands to help open the airway of a patient?” and then through directed instruction to the participant if the scripted prompt did not lead to corrective action.

### Scoring

Numerical scoring was performed solely for the purposes of validity testing of this formative tool. The two raters independently rated the individual performance of all 12 subjects in real time using the observational PAAT checklist, rating items as *0* if subjects did not successfully complete the task without additional prompting, or *1* if subjects completed the task successfully on their first attempt. For the purpose of this pilot study and validity testing, only participants' first attempts were scored. Final scores were the average of the two raters' scores across items. Three summary scores were reported. The total basic score included scores for basic airway maneuvers, airway adjunct use, and bag-valve mask ventilation. The total advanced score included advanced airway equipment preparation, direct laryngoscopy, and video laryngoscopy. The overall skill score was the sum of basic and advanced scores.

For use of this formative assessment tool in practice, it was decided that in order to successfully finish the activity, the participant would have to successfully complete the entire checklist. Participants would be required to correct their errors if they inserted any adjunct in a manner that would be dangerous to a patient. Our group devoted considerable time to the issue of appropriate context for use of PAAT, which ultimately was decided by consensus of our expert panel. Because our intended use was to measure the basic pediatric airway skills such that a learner would be able to perform under direct supervision, we determined that the standard should allow for coached performance. We trained our raters to provide real-time prompts, followed by immediate feedback and one-step remediation if a learner was unsuccessful at any step. However, we did agree as a group that because airway management is such a critical skill and that failure carries life-threatening consequences, each of the steps on the PAAT checklist was critical to safe and effective performance and that inability to achieve any of the steps after real-time remediation would require substantial reteaching of the skills. In this sense, then, failure to perform any step of the process could be deemed failure of the process. Trainees who were unable to complete the task even with coaching would be offered additional training and support on an individual basis.

### Statistical Analyses

Each rater scored each checklist component as present (1) or absent (0). The scores from each rater across participants were summed, and the average scores for basic, advanced, and overall airway skills between the two raters were used in the analyses to assess differences in scores as a function of experience level across the four groups (medical students, residents, pediatric emergency medicine fellows, and pediatric emergency medicine attending physicians). Scores were used to calculate agreement rates and interrater reliability. Data were analyzed using SAS Software (version 9.4). Comparisons between the groups were evaluated using the Kruskal-Wallace, and the Jonckheere-Terpstra test assessed the trend in increasing differences between the experience groups.

The agreement between raters for the three summary scores was calculated in three ways. First, a Pearson correlation coefficient for each overall skill score and the composite scores for the basic and advanced totals was calculated. Second, a Cohen's Kappa (Κ) coefficient was calculated as a measure of interrater reliability. Finally, a Kappa squared (Κ^2^), was used to provide an estimate of the accuracy (by the percentage of variance estimated by the agreement between raters) of these scores.^20^ Cohen's Kappa and Kappa^2^ were calculated only for the summary scores and not the basic or advanced scores, as the restricted variance within the composite elements would likely inflate the Kappa values. The 95% confidence interval on rater agreement was also reported.

## Results

### Participant Characteristics

There was a broad range of prior pediatric airway experience ranging from students (no prior experience) to experts. Pediatric emergency medicine fellows and attending physicians had more experience in most domains, notably in that of video laryngoscopy, where no medical students or residents had any prior experience in this skill.

### Validity Evidence

The PAAT was designed to provide a comprehensive formative assessment for resident pediatric airway skills performance. The process of collecting and interpreting validity evidence to support reliance on an assessment's decision was fundamental to its use.^21^ Sources of validity evidence were summarized in Table 1.^22^ Kane's framework identifies four inferences used to support the validity argument: scoring, generalization, extrapolation, and implications.^23–25^ The validity argument rested on the intended use of the assessment tool. Our tool was intended to measure and provide feedback to learners such that a resident would be sufficiently prepared to perform these skills under supervision in clinical practice.

**Table 1.**
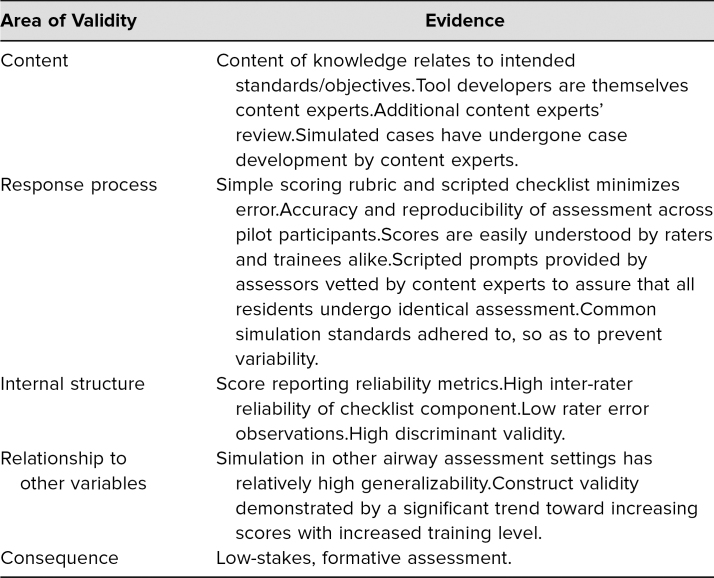
Sources of Validity Evidence for the Proposed Pediatric Airway Assessment Tool

The PAAT was intended to generate reproducible, accurate scoring through use of scripted instructions, clear checklist items, and a simple scoring rubric. Generalization—using a score as a reflection of actual performance in a test setting—was demonstrated with strong interrater reliability.^26^ The correlation between raters' evaluation of the elements within each summary score were high, ranging from 0.92 (adolescent bagging rate) to 1 (basic airway maneuvers) (Table 2). Interrater reliability (K) across the summary scores total was high (0.92 to 1). The Κ^2^ for each summary score (1 total basic score; 0.83 total advanced score; 0.85 total score) indicated little to no variance in the observed airway skills performance due to disagreement between the raters.

**Table 2.**
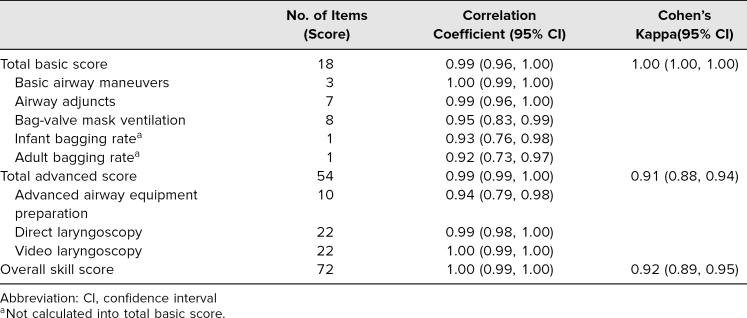
Scoring Comparison Between Raters

Extrapolation of scores to be used as a reflection of real-world performance was supported through demonstrating content evidence provided by expert panel review and derivation from the PALS curriculum,^19^ demonstrating selection of appropriate subject matter. Evidence supporting the claim that the tool reflected the intended construct was demonstrated by the observed variation in scores by experience level; those who have demonstrated real-life experience (fellows and attendings) scored higher on the tool than those with little or no clinical experience (medical students and residents) (Table 3). As seen in Table 3, there was a significant effect of experience level on the knowledge component and in each pediatric airway score (*p* = .03). For the procedural scores, there was also an ordering effect, with higher scores seen with increased levels of training (*p* = .003), with most differences noted between fellows/attendings (i.e., experienced) and residents/medical students (i.e., inexperienced). Fellows and attending physicians outscored medical students and residents in all domains. Fellows and attending physicians had similar scores for knowledge and performance on basic airway skills and overall score, and fellows had the highest total advanced airway score.

**Table 3.**
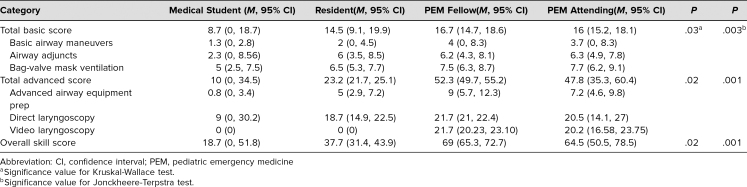
Pediatric Airway Assessment Scores (*n* = 12, 95% Cl)

## Discussion

Formative assessment-based frameworks focus on training for physician performance and patient care outcomes, emphasizing assessment and correction of observed, discrete, measurable behaviors.^27–29^ The ACGME has established benchmarks, which include the assessment of proficiency in airway management.^10^ However, developing rigorous assessment tools remains challenging. Tools should be reliable and predictive of future success, with clearly defined outcomes so that observers can make objective judgments.^30^ Tools have been published to assess procedural skills including central venous catheter insertion,^31^ pediatric resuscitation,^14^ and lumbar puncture.^32,33^ However, validity evidence for tools to assess pediatric airway skills performance is lacking. Therefore, we designed the PAAT as a formative assessment to prepare learners in their progress towards competency in these skills. We, refined it based on expert feedback and prior airway checklists, and then piloted it to collect and interpret validity evidence for its future use.^14–17^ The tool may be modified into two separate tools—one for basic airway assessment and one for advanced airway assessment—depending on the intended use of the tool. For the purposes of scoring and testing the tool, we only scored the first pass, but we recommend rapid cycle repetition and allowing several attempts as an educational effort with future uses.

One interesting finding was that fellows scored higher than attendings in the advanced airway score. The reason for this difference was likely multifactorial. One possibility included the fact that the fellows had more recent training on, and experience with, advanced airway skills, particularly video laryngoscopy. These assessments strengthened the inference that PAAT scoring ought to reflect performance in real-life scenarios. We believe that the strength of the scoring, generalization and extrapolation inferences supported further investigation of the tool's use in assessment of pediatric airway skills performance.

We presented several aspects of validity evidence for our tool which was piloted and is expected to be used for training in the simulation environment. With any assessment tool, there are likely to be threats—anticipated or unanticipated—to the validity inferences between observed performance in the test setting and the unobserved proficiency in the setting of actual practice. While we sought to minimize the impact of these threats, their potential must be acknowledged. Not included in the current version of the PAAT is a separate knowledge assessment. It is likely that there are certain discrete knowledge items (e.g., appropriate depth of tube insertion) that could be assessed using a multiple-choice questionnaire or other format.

There were a number of limitations to our tool and this study. The relatively small size of the pilot sample was a clear limitation. While we detected significant differences among training groups, larger studies should determine more clearly how the tool performs, particularly for learners away from the extremes of experience. Additionally, the volunteerism of the pilot sample may select for more conscientious participants, which may actually affect skill set. The use of raters who were part of the development team, not blinded to the subjects' training level, could introduce bias and limit the interpretation of inter-rater reliability. However, this will be likely during routine use of this tool at any institution. To standardize the assessment and minimize bias, each scenario was tightly scripted to ensure consistency. Checklist items were observable, objective, yes/no questions. Still, the tool may not perform as well if used by educators who have not been as extensively trained. Due to the use of a convenience sample, selection bias may have occurred, though participants were selected based only on availability, which should have no bearing on pediatric airway skill set. The use of simulation versus live patients for assessment is necessary but does limit extrapolation to live clinical performance. If the physical, emotional, or conceptual realism of the scenario were not maintained, this may affect the response process and reliability of the tool. We cannot comment on the ability of this assessment to predict performance in the clinical setting based on performance in the simulated setting. This should be addressed in future studies.

### Conclusion

Formative assessment tools are a crucial component to address the mandate to assess patient care procedural skills during residency training. Such tools provide objective measurements of resident procedural skills that can be used by programs to enhance training by tracking resident progress over time and guiding specific changes in program curricula. Prior to the development of this resource, there existed no validated formative assessment tool for training in comprehensive pediatric airway skills performance. This publication provides both an easily implemented assessment tool for training and initial evidence to support validity of its use.

## Appendices

Pediatric Airway Skills Assessment Checklist.docx
All appendices are peer reviewed as integral parts of the Original Publication.
