# Supplementary material for: Pediatric Airway Assessment Tool (PAAT): A Rating Tool to Assess Resident Proficiency in Simulated Pediatric Airway Skills Performance
Source: MedEdPORTAL. 2020 Oct 19;16:10997. doi: 10.15766/mep_2374-8265.10997 (PMC7586756; doi:10.15766/mep_2374-8265.10997)
Supplement: Supplementary file 1 — Pediatric Airway Skills Assessment Checklist.docx [file mep_2374-8265.10997-s001.zip › A. Pediatric Airway Skills Assessment Checklist.docx]

**VENTILATORY MANAGEMENT SKILLS**

| - Instruct learners to verbalize all actions - If a learner does not successfully complete each of the items in a section, please immediately remediate the student prior to proceeding to the next section - If learner is unable to successfully complete maneuver with prompting after 3 attempts in a way that is not dangerous to the patient, please highlight this item (as this will mean failure of the proficiency assessment and need for remediation). | **✓**  **1st Attempt** | **✓**  **Successfully Complete after 1^st^ Attempt** |
| --- | --- | --- |
| ***Basic Airway Maneuvers***  *Prompt:* “*Demonstrate how to properly position and open the airway of a patient presenting in respiratory distress” If the student does not perform all maneuvers, prompt: “Is there anything else that you can do with your hands to help open the airway of a patient?”* | | |
| Opens airway manually using **jaw thrust** |  |  |
| Opens airway manually using **head tilt with chin lift** |  |  |
| Uses appropriately-placed towel roll:   - Towel roll placed under shoulders in infant |  |  |
| ***Airway Adjuncts***  *Display all sizes of Nasopharyngeal Airways (NPAs) and Oropharyngeal Airways (OPAs).*  *Ask the student to explain the indications and contraindications for use of each airway adjunct. Then ask the student to place an appropriately sized NPA and OPA* into the manikin.* | | |
| Prompt: “Verbalize an indication for airway adjunct use.”   - Risk of obstruction due to relaxed upper airway muscles or tongue (examples include post-ictal patient, intoxicated patient, cardiac arrest patient) |  |  |
| Prompt: “Verbalize 2 contraindications for nasopharyngeal airway (NPA) usage.”   - Suspected head trauma/maxilla fracture - Active epistaxis |  |  |
| Prompt: “Select and place a correctly sized NPA”:  Correctly-sized NPA* chosen by measuring from tip to nose to tragus of ear |  |  |
| Inserts NPA correctly |  |  |
| Prompt: “Verbalize 2 contraindications for oropharyngeal airway (OPA) usage.”   - Intact gag reflex - Oral trauma (ex. Broken teeth) |  |  |
| Prompt: “Selects and place a correctly sized OPA”:  Correctly-sized OPA* chosen by measuring from corner of mouth to angle of mandible |  |  |
| Inserts OPA correctly |  |  |
| ***Bag-Valve Mask Ventilation***  *Display multiple sizes of mask and bags.*  *Prompt: “Select the proper size mask and bag* for ventilation of both adolescent and infant manikins. Then, demonstrate effective bag-mask ventilation with an appropriate rate and volume.”* | | |
| Selects correct mask and bag size for ventilations |  |  |
| Connects bag to oxygen source |  |  |
| Applies bag-mask device and opens airway using E-C clamp technique |  |  |
| Ventilates with BVM for 1 minute with adequate rate & chest rise in **FOUR scenarios**:  (Allow learner to complete 1 full minute of BVM while counting how many breaths given in that minute and assessing for chest rise. Record this information in chart below for single provider bagging. After 1 full minute, you may correct technique or rate if necessary.) |  |  |
| 1. **Infant** / **Single** **provider** technique |  |  |
| 1. **Infant** / **Two** **provider** technique (instructor to bag while learner holds mask) |  |  |
| 1. **Adolescent** / **Single** **provider** technique |  |  |
| 1. **Adolescent** / **Two** **provider** technique (instructor to bag while learner holds mask) |  |  |
| Troubleshoots patient with poor chest rise (either infant or adolescent)   - Prompt: “You are now having difficulty with bagging the patient. Perform and verbalize any necessary maneuvers to optimize your airway management.” - Students should reposition airway and insert an airway adjunct. |  |  |

|  | ***Target Rate*** | **Actual Rate** | **Chest rise (Y/N)** |
| --- | --- | --- | --- |
| **Infant 1 Provider** | *12-20 bpm (q 3-5 sec)* |  |  |
| **Adolescent 1 Provider** | *10-12 bpm (q 5-6 sec)* |  |  |

**INFANT ADVANCED AIRWAY SKILLS**

| - Instruct learners to verbalize all actions - If a learner does not successfully complete each of the items in a section, please immediately remediate the student prior to proceeding to the next section - If learner is unable to successfully complete maneuver with prompting after 3 attempts in a way that is not dangerous to the patient, please highlight this item (as this will mean failure of the proficiency assessment and need for remediation). | **✓**  **1^st^ Attempt** | **✓**  **Successfully Complete after 1^st^ Attempt** |
| --- | --- | --- |
| ***Airway Equipment Preparation***  *Start with bed lowered. Display multiple sizes of endotracheal tubes (ETTs). Students are allowed to use PALS card. If asked, weight is 8 kg.*  *Prompt: “Prepare to intubate this infant. Verbalize what equipment you would like to ensure is available and functioning prior to starting the procedure. Medications are available and drawn up already.”* | | |
| States all equipment needed for procedure (blade, ETT, stylet, syringe, suction, oxygen, end-tidal/exhaled CO_2_ device)   - If learner misses one or more items, tell them the items and ask them again to “verbalize what equipment you would like to ensure is available and functioning prior to starting the procedure”. |  |  |
| Selects proper size endotracheal tube (ETT) (3.5 – 4.0 cuffed ETT) |  |  |
| Check ETT cuff by inflating and deflating balloon with syringe |  |  |
| Verbalize if difficult airway is anticipated and ensure backup methods are available |  |  |
| Ensures proper patient positioning (ex. Height of bed) |  |  |
| ***Direct Laryngoscopy - Infant***  *Display multiple sizes of Mac and Miller blades.*  *Prompt: “Prepare for direct laryngoscopic intubation. Then, intubate while verbalizing your technique out loud.”* | | |
| Selects proper size laryngoscope blade (Miller 1) |  |  |
| Assembles laryngoscope properly and tests blade light source |  |  |
| Inserts stylet into ETT correctly |  |  |
| Holds laryngoscope in left hand |  |  |
| Inserts blade in correct fashion (sweeping tongue from right to left and gently advancing) |  |  |
| Elevates mandible using laryngoscope without pivoting on teeth |  |  |
| Inserts ETT through vocal cords |  |  |
| Inserts ETT to proper depth (3 x tube size at teeth/gums) |  |  |
| Inflates ETT cuff and disconnects syringe |  |  |
| Remove stylet from ETT |  |  |
| Prompt: “How do you confirm that the ETT is in proper location?” Confirms placement of ETT by visualization, exam (chest rise and auscultation), AND by using exhaled CO_2_ device |  |  |

**ADOLESCENT ADVANCED AIRWAY SKILLS**

| - Instruct learners to verbalize all actions - If a learner does not successfully complete each of the items in a section, please immediately remediate the student prior to proceeding to the next section | **✓**  **1^st^ Attempt** | **✓**  **Successfully Complete after 1^st^ Attempt** |
| --- | --- | --- |
| ***Airway Equipment Preparation***  *Start with bed lowered. Display multiple sizes of endotracheal tubes (ETTs). Students are allowed to use PALS card. If asked, weight is 60 kg.*  *Prompt: “Prepare to intubate this adolescent. Verbalize what equipment you would like to ensure is available and functioning prior to starting the procedure. . Medications are available and drawn up already.””* | | |
| States all equipment needed for procedure (blade, ETT, stylet, syringe, suction, oxygen, end-tidal/exhaled CO_2_ device)   - If learner misses one or more items, tell them the items and ask them again to “verbalize what equipment you would like to ensure is available and functioning prior to starting the procedure”. |  |  |
| Selects proper size endotracheal tube (ETT) (7.0 – 7.5 cuffed ETT) |  |  |
| Check ETT cuff by inflating and deflating balloon with syringe |  |  |
| Verbalize if difficult airway is anticipated and ensures backup methods are available |  |  |
| Ensures proper patient positioning (ex. Height of bed) |  |  |
| ***Video Laryngoscopy - Adolescent***  *Display multiple sizes of Glidescope blades. Adolescent size Glidescope wand should already be hooked up, but machine should be off.*  *Prompt: “Prepare for video laryngoscopic intubation. Then, intubate while verbalizing the technique out loud.”* | | |
| Selects proper size Glidescope blade (Size 3 or 4) |  |  |
| Inserts stylet with appropriate curvature to match Glidescope blade |  |  |
| Holds Glidescope blade in left hand |  |  |
| Looks into the ***mouth*** to insert the blade at midline |  |  |
| Looks at the Glidescope ***screen*** to obtain the best view of the glottis on the screen |  |  |
| Looks at the ***mouth*** to introduce the ETT into right side of mouth |  |  |
| Looks at the Glidescope ***screen*** to guide the ETT through the glottis |  |  |
| Pull back stylet slightly from ETT to assist in advancing through the vocal cords |  |  |
| Inserts ETT to proper depth (3 x tube size at teeth/gums) |  |  |
| Inflates ETT cuff and disconnect syringe |  |  |
| Prompt: “How do you confirm that the ETT is in proper location?” Confirms placement of ETT by visualization, exam (chest rise and auscultation), AND by using exhaled CO_2_ device |  |  |
| ***Direct Laryngoscopy - Adolescent***  *Display multiple sizes of Mac and Miller blades.*  *Prompt: “Prepare for direct laryngoscopic intubation. Then, intubate while verbalizing the technique out loud.”* | | |
| Selects proper size laryngoscope blade (Mac or Miller 3 or 4) |  |  |
| Assembles laryngoscope properly and tests blade light source |  |  |
| Inserts stylet into ETT correctly |  |  |
| Holds laryngoscope in left hand |  |  |
| Inserts blade in correct fashion (sweeping tongue from right to left and gently advancing) |  |  |
| Elevates mandible using laryngoscope without pivoting on teeth |  |  |
| Inserts ETT through vocal cords |  |  |
| Inserts ETT to proper depth (3 x tube size at teeth/gums) |  |  |
| Inflates ETT cuff and disconnects syringe |  |  |
| Remove stylet from ETT |  |  |
| Prompt: “How do you confirm that the ETT is in proper location?” Confirms placement of ETT by visualization, exam (chest rise and auscultation), AND by using exhaled CO_2_ device |  |  |

***CRITERIA FOR SUCCESSFUL COMPLETION:***

_____ 100% of checklist completed

_____ Does not insert any adjunct in a manner that is dangerous to the patient

| **Course Passed: Y N**  **Reason for Failure:_________________________________________________________________**    **Signature: ___________________________________** |
| --- |

***Appropriate Manikin Equipment Sizing Chart**

|  | **Sim Baby** | **Sim Man** |
| --- | --- | --- |
| **Bag** | Infant/Child | Large |
| **Mask** | Infant or Toddler | Medium |
| **OPA** | 6 cm | 8 cm |
| **NPA** | 3.5 cm | 7.0 cm |
| **Endotracheal Tube** | 3.5 – 4.0 | 7.0 – 7.5 |
| **Direct Laryngoscopy Blade** | Miller 1.0 | Mac/Miller 3 or 4 |
| **Video Laryngoscopy Blade** | 2 | 3 or 4 |
